# The Fata Morgana of Unconscious Perception

**DOI:** 10.3389/fnhum.2018.00120

**Published:** 2018-04-10

**Authors:** Marjan Persuh

**Affiliations:** Department of Social Sciences, Human Services and Criminal Justice, Borough of Manhattan Community College, City University of New York, New York, NY, United States

**Keywords:** visual perception, unconscious perception, vision, visual processing, visual cortex

## Introduction

The human nervous system contains numerous visual pathways, suggesting that visual information is represented differently for different purposes. For example, an influential view holds that two separate but interacting processing streams result in vision for action and vision for perception (Milner and Goodale, 1995). Although much evidence in support of this view comes from patient studies (Milner et al., 1991), studies with healthy participants suggest that processing of visual information for perception and action (e.g., eye movements) can be dissociated (Spering et al., 2011). Most studies of perception use stimuli that are consciously perceived; however, the possibility of unconscious perception continues to intrigue the research community. In this paper I critically examine a large body of evidence for unconscious perception in healthy human participants obtained through a variety of methods that render stimuli invisible (Kim and Blake, 2005; Breitmeyer, 2015). Most researchers in the field have not been convinced by few authors skeptical about unconscious perception (Dulany, 1997; Holender and Duscherer, 2004; Peters et al., 2017). Here I question evidence for unconscious perception based on some general principles of studying mechanisms of biological pathways.

I do not address studies on patients with disorders of awareness such as blindsight, neglect, and visual agnosias. It is difficult to establish the absence of phenomenology from patients' reports because of response bias. Additionally, there is always a possibility that brain damage affects patients' reports of conscious perception. Furthermore, some recent studies cast doubt on the absence of phenomenology in these patients. For example, a recent study that used a graded measure of awareness, suggests that stimuli in blindsight patients might in fact be weakly consciously perceived (Mazzi et al., 2016).

Unconscious perception has been purportedly demonstrated in a number of studies (for excellent reviews see Kouider and Dehaene, 2007; Faivre et al., 2014; Sterzer et al., 2014; Yang et al., 2014). These studies have been plagued with methodological problems that still linger and repeatedly cast doubt on the validity of the phenomenon itself. Here, I suggest that we should focus on a different question. Sidestepping for the moment issues with measuring awareness, what can we say about unconscious perception?

## How powerful is unconscious perception?

The possibility of unconscious perception has intrigued scholars since the beginning of experimental psychology (Peirce and Jastrow, 1884; Sidis, 1898). By using masking to prevent conscious perception of the stimulus, Marcel was the first to demonstrate unconscious semantic priming, in which a semantically related prime affects processing of the target word (Marcel, 1983b). He proposed that unconscious perception redescribes sensory data to the highest possible level of description available to the organism (Marcel, 1983a). More recently, some authors have proposed that above-chance performance in a perceptual discrimination task in the absence of stimulus awareness provides strong evidence for unconscious perception (Peters and Lau, 2015). To agree on what constitutes evidence for unconscious perception, a definition of perception is needed. Several definitions have been proposed. To dissociate perception from consciousness, it has been suggested that perception involves extraction of perceptual information from a stimulus (Kanwisher, 2001). For example, when viewing an ambiguous Necker cube, the percept alternates between two states, yet the stimulus itself does not change. Others have further suggested that the definition of perception includes perceptual constancies (Burge, 2010). It has been also proposed that to count as perception, such representations should be available for the control and guidance of action (Dretske, 2006). Although there is no universally accepted definition, I will, for the purposes of this paper, adopt a definition of perception that has in similar form been suggested before (Kanwisher, 2001; Dretske, 2006; Burge, 2010) and discuss studies that meet this definition. By perception, I mean the extraction of perceptual information (including perceptual constancies) that is represented at the personal or more broadly, organismal (individual) level and can be used for the control and guidance of action. For example, suppose that the word CLAP is perceived unconsciously and prompts observer to clap. That would be a clear example of unconscious perception; I return to this example later when I compare conscious and unconscious perception.

An important set of results, relevant to the issue of unconscious perception, comes from the electroencephalography (EEG) studies on automatic change detection. Our nervous systems represent statistical regularities and automatically detect novel or surprising events. This phenomenon is known in the auditory domain as mismatch negativity (MMN; vMMN in the visual domain) (for a review see Stefanics et al., 2014) and the neural system generating MMN is considered a primitive system of intelligence (Näätänen et al., 2001). Considered a perceptual prediction error, vMMN can be elicited even by changes in complex stimulus attributes, such as facial emotions (Susac et al., 2004; Zhao and Li, 2006) or laterality of body parts (Stefanics and Czigler, 2012). It is well known that MMN can be elicited even in sleep (Nashida et al., 2000; Atienza and Cantero, 2001; Stefanics et al., 2007, 2009) or in comatose patients (Kane et al., 1993, 1996; Fischer et al., 1999), suggesting that complex visual information processing occurs in the absence of awareness, although some recent studies suggest that perceptual awareness might be necessary for vMMN generation (Flynn et al., 2017). Because most vMMN studies do not collect behavioral data, we currently do not have evidence that visual processing indexed by MMN can guide action and this type of processing currently does not meet the definition of perception adopted in this paper. Similarly, other studies that report only differences in neural activity, do not provide evidence for unconscious perception. We now turn to evidence for unconscious perception from studies that employ a dissociation paradigm.

Various paradigms have been developed to demonstrate unconscious perception (reviewed in Merikle et al., 2001). The majority of studies of unconscious perception employ a dissociation paradigm, which in the most general form requires dissociation of two measures, a measure of conscious perception and a measure of unconscious perception (Schmidt and Vorberg, 2006). The most popular is a simple dissociation paradigm, which requires demonstration of unconscious perception in the absence of conscious perception, typically achieved by the bottom-up suppression of target stimuli.

For studies with healthy subjects, researchers can choose from a variety of methods to render stimuli invisible (Kim and Blake, 2005; Breitmeyer, 2015). Most studies however employ some form of visual masking in which a mask follows a brief stimulus presentation, preventing its conscious perception (Breitmeyer and Ögmen, 2006). Unconscious perception of the stimulus is then demonstrated through an indirect effect such as priming, in which a prime stimulus affects a response to the subsequently presented target. Masked priming studies provided evidence for unconscious perception of simple stimulus features such as color or shape (Neumann and Klotz, 1994; Breitmeyer et al., 2004; Ro et al., 2009). Using masking as well as other approaches that allow for longer stimulus presentation times such as continuous flash suppression (CFS) (Tsuchiya and Koch, 2005) or crowding (Korte, 1923; Flom et al., 1963; Bouma, 1970), more complex stimulus features such as facial identity and emotional expression have been demonstrated to occur unconsciously (reviewed in Axelrod et al., 2015). Climbing the complexity ladder, we have strong evidence for unconscious semantic priming (Dehaene et al., 1998; Naccache and Dehaene, 2001) and even unconscious inhibition, which is a form of cognitive control previously believed to require awareness (van Gaal et al., 2008, 2010, 2012). Taken together, the evidence seems to suggest that most of our perception, if not all, can occur unconsciously.

Presented this way, unconscious perception seems to have few limits, suggesting that the role of consciousness might be minor. Furthermore, to prevent conscious perception, researchers use one of the many techniques that are available (Kim and Blake, 2005; Breitmeyer, 2015). Because each method affects unconscious perception to a different degree, every unconscious perception is unconscious in its own way. It is possible that with a different or improved method, the limits of unconscious perception will be further expanded.

## Every unconscious perception is unconscious in its own way

Because multiple paradigms are rarely used within one study, conclusions about unconscious perception are usually based on one experimental paradigm. In masking studies for example, a stimulus is presented only for tens of milliseconds and then masked with another stimulus of longer duration. Failure to demonstrate unconscious perception might be attributed to limited stimulus processing. Although some other techniques, such as CFS (Tsuchiya and Koch, 2005), allow for longer presentation times, stimulus processing is strongly affected by simultaneous presentation of noise to one eye, while stimulus is presented to the other eye.

Some studies directly compared different experimental paradigms using the same stimuli. For example, Fogelson et al. (2014) collected fMRI data while subjects viewed images of faces and tools. Images were rendered invisible using either CFS or chromatic flicker fusion (CFF), in which two oppositely colored images are presented to both eyes, at a frequency that results in image fusion and perception of uniform color. Category information about CFS suppressed stimuli was recovered only from the occipital cortex, whereas category information about CFF suppressed stimuli, was also recovered from temporal and frontal regions, suggesting that stimuli are processed differently under different suppression methods. Although neural activity by itself is not evidence for unconscious perception, this study demonstrates important methodological constraints.

These considerations seem to allow two important conclusions. First, it is premature to conclude that a specific stimulus cannot be perceived unconsciously if only one experimental approach has been tested. Second, stimuli for which unconscious perception has not been demonstrated might still be perceived unconsciously with an improved or novel paradigm that would eliminate conscious perception but have minimal effect on unconscious perception.

I will now propose that the approach of using different experimental paradigms to interfere with visual processing, leading to conscious perception of a stimulus, can be compared to methodology of interfering with cellular pathways leading to a specific end product. I chose as an example, bacterial signal transduction leading to formation of a dormant structure, called a spore (Hilbert and Piggot, 2004), but any other complex biological process would serve just as well. When viewed from this perspective, very different conclusions can be made about the role of unconscious perception.

## Disrupting cellular pathways vs. disrupting visual processing

When faced with environmental stress, bacterium *B. subtilis* has at its disposal a variety of responses; the most extreme form of stress adaptation is the formation of a dormant, heat and radiation resistant, multilayered spore (Hilbert and Piggot, 2004). Sporulation pathway can be examined by disrupting different processing stages. One classical approach is to use mutagenesis, which affects cells at the level of DNA. Using mutagenesis, the formation of the spore can be modified or completely prevented. Each mutation leaves the upstream processing intact; in other words, even when cells fail to produce spores, parts of regulatory network remain active. In this analogy, the sporulation process corresponds to visual processing and a complete spore as the end product corresponds to a conscious percept in vision. An unconscious percept, which is considered to have all of the qualities of a conscious percept except for observers not being conscious of it, corresponds to a spore with all of its components except for the outermost layer.

How should we best describe processes that occur in sporulation mutants? When masking a briefly presented visual stimulus, we sometimes describe our visual experience as partial awareness (Kouider et al., 2010). In the absence of awareness, any visual processing that is preserved and measured through indirect measures such as priming, is described as unconscious or subliminal perception. Sporulation researchers feel no need to invent new terms to describe processing in the absence of a complete spore as the end product. Mutations simply affect the sporulation process at a specific stage. To make the comparison to visual perception even more concrete, let us consider one of the techniques used for visual suppression, called motion-induced blindness (MIB) (Bonneh et al., 2001). In MIB, a rotating mask intermittently suppresses conscious perception of a target stimulus. It has been demonstrated that target-specific suppression occurs specifically in visual area V4; in no other early visual areas (V1–V3) is suppression significant (Donner et al., 2008). The role of the rotating mask in suppressing the target in MIB is analogous to the role of a mutation in preventing sporulation. It seems most parsimonious then to conclude that different techniques that prevent conscious perception of stimuli simply interfere with visual processing at a specific level. What remains is just residual visual processing. Do we have any reason to call that residual visual processing unconscious perception? To answer this question, we need to examine the effects of putative unconscious perception at the level of the organism a little closer.

## Visual perception at the level of the organism

Visual perception evolved to guide successful behavior and to promote survival. Conscious visual perception allows us to perform complex tasks and to explore our environment. How does unconscious perception compare? Let's examine a very simple, hypothetical task, in which a participant is presented with a single word and instructed to perform the action specified by it. Words are presented in isolation or masked. Let's say that the first non-masked word is STAND. We don't expect any difficulty for the participant to follow the instructions; no one would be surprised to see the participant stand up. Now imagine that next, the masked word is CLAP. Because the word is masked, it is not visible to participant; however it should be processed and perceived unconsciously. What do we expect? If the participant suddenly starts clapping, we would be amazed. Indeed, there is no evidence that such a simple task can be performed if the word is perceived unconsciously.

We are left with the effect of unconsciously perceived stimuli on reaction times and accuracy in priming studies. We therefore have evidence for unconscious perception that is mostly based on modulation of reaction times in a highly constrained and limited tasks. From this perspective a very different picture emerges, far from being powerful and rich, unconscious perception seems very limited and weak at best.

## Conclusions and implications for vision research

Always controversial, unconscious perception has been proposed to be powerful and rich. However, upon closer inspection, it becomes evident that we can't do much with stimuli perceived unconsciously. Demonstrations of unconscious perception are based on paradigms that interfere with visual processing and can be compared to other studies that disrupt cellular networks, such as the sporulation process in bacteria. Cellular manipulations of the sporulation network prevent formation of spores, yet leave parts of the sporulation process intact. By analogy, the most parsimonious conclusion is that the effect of putative unconscious perception on our behavior only represents residual visual processing.

If we accept the idea that putative unconscious perception is in fact residual visual processing, what are the implications for vision research? Should we abandon research on unconscious perception? A large part of the enterprise of discovering neural correlates of consciousness is based on comparing differences in neural activity between unconscious and conscious perception. Does accepting the proposed idea invalidate this paradigm? I think the answer to both questions is no.

Just as mutagenesis is an extremely useful tool for uncovering the architecture of cellular pathways, masking and other paradigms can reveal something about the architecture of visual processing. As we explore in more detail the mechanism and levels at which different suppression techniques disrupt visual processing, we can learn about the process that results in conscious visual perception. Ideally, we might be able to demonstrate and characterize different processing stages, predicted by cognitive and computational models. At the same time, we should be careful to avoid the temptation to label such processing unconscious perception, unless we have good independent reasons to do so.

## Author contributions

The author confirms being the sole contributor of this work and approved it for publication.

### Conflict of interest statement

The author declares that the research was conducted in the absence of any commercial or financial relationships that could be construed as a potential conflict of interest.
